# Post-harvest biocontrol of *Fusarium* infection in tomato fruits using bio-mediated selenium nanoparticles

**DOI:** 10.1186/s13568-023-01622-y

**Published:** 2023-10-23

**Authors:** Howaida M. Manaa, Ebtsam M. Hamza, Noha M. Sorour

**Affiliations:** 1 Department of Plant Biotechnology, Genetic Engineering and Biotechnology Research Institute, University of Sadat City, 22857/79, Sadat City, Egypt; 2 Department of Industrial Biotechnology, Genetic Engineering and Biotechnology Research, Institute, University of Sadat City, 22857/79, Sadat City, Egypt

**Keywords:** Bioagent, Selenium, *Fusarium*, Tomato, Post-harvest

## Abstract

The protection of post-harvest infection by *Fusarium* spp. is a major worldwide demand, especially using effective natural alternatives to chemical fungicides. In this respect, selenium nanoparticles (SeNPs) were biosynthesized using Fenugreek seeds aqueous extract. Bio-mediated SeNPs were characterized using XRD, FTIR, UV–Vis, TEM, and EDX. XRD confirmed the crystalline nature with six characteristic peaks corresponding to Se-nanocrystals. TEM showed spherical-shaped SeNPs with 34.02–63.61 nm diameter. FTIR verified the presence of different bio-functional groups, such as, N–H, O–H, C–N, and C–NH_2_ acting as stabilizing/reducing agents during the biosynthesis. Bio-mediated SeNPs exhibited excellent biocidal activity against *F. oxysporum* and *F. moniliforme*, with MIC of 0.25 and 1.7 mg/mL, respectively. Direct treatment of *F. oxysporum* with SeNPs led to significant deformation and lysis of the fungal hyphae within 18 h. The treatment of infected fruits with MIC of SeNPs reduced the infection signs by 100% and preserved the fresh-like appearance of treated fruits for 25 and 35 days when stored at 25 °C and 5 °C, respectively. Therefore, SeNPs is considered efficacious fungicidal against *Fusarium* spp. in-vitro and in-vivo. The treatment of tomato fruits with MIC of SeNPs positively affected its chemical properties, as well as decreased weight loss %, confirming the barrier effect of SeNPs, thus increasing fruits’ shelf-life. Bio-mediated SeNPs appeared safe towards normal HSF and OEC cell lines with IC_50_> 300 μg/mL. Overall results recommend the usage of bio-mediated SeNPs as safe powerful bioagent against *Fusarium* infection, maintaining tomato quality, and providing protection from post-harvest invasion and/or destroying existing infections.

**Graphical Abstract:**

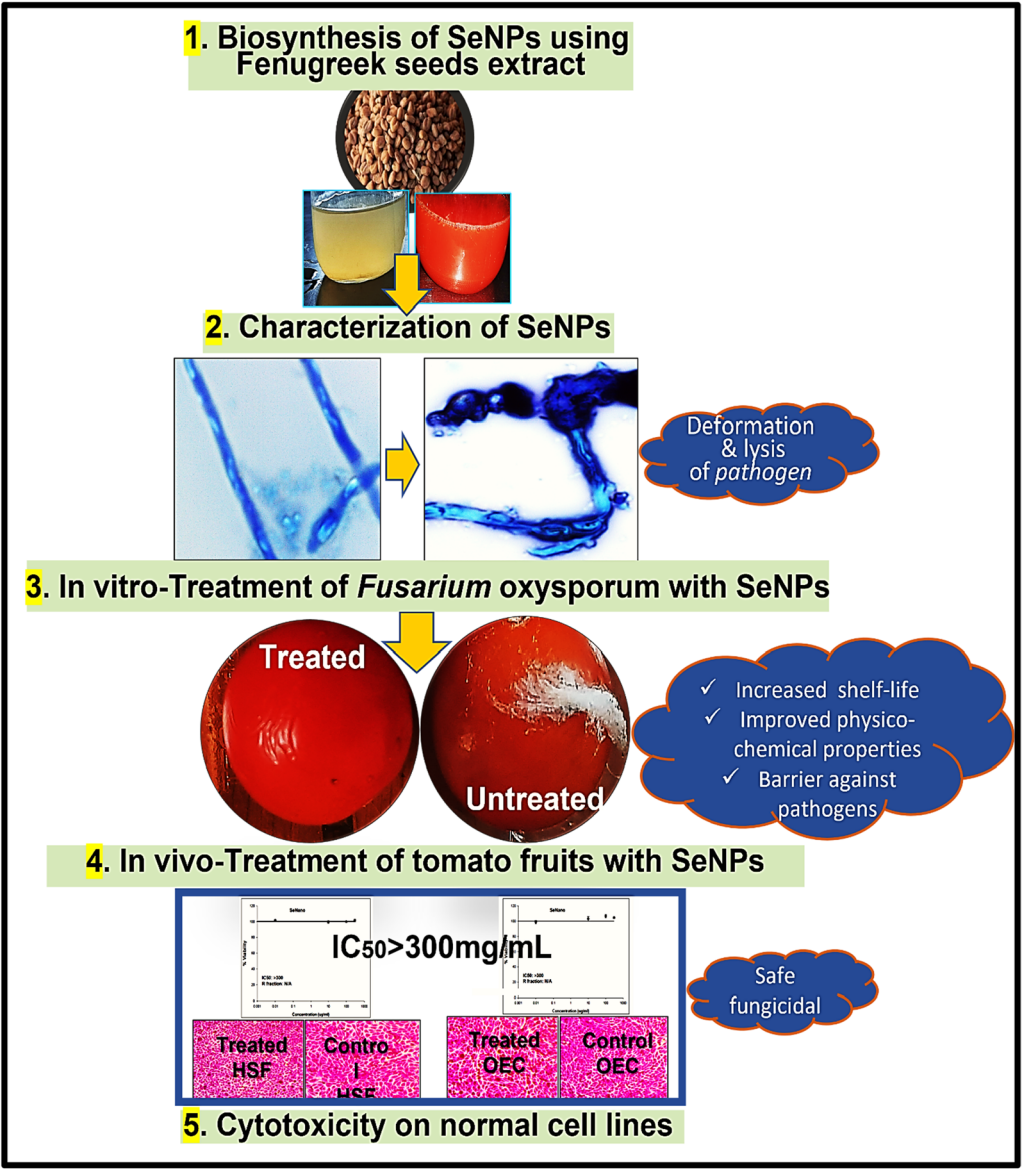

## Highlights

- Bio-mediated SeNPs by Fenugreek seeds extract exhibited excellent biocidal activity against two *Fusarium* spp.
- Treatment of *F. oxysporum* mycelia with SeNPs led to significant lysis.
- Treatment of infected fruits with MIC of SeNPs reduced infection signs by 100%.
- Bio-mediated SeNPs appeared safe towards normal HSF & OEC cell lines with IC_50_> 300 μg/mL.
- SeNPs maintained the quality of tomato & provided protection from post-harvest fungal invasion and/or destroying existing infections.

## Introduction

One of the most frequently grown plants is tomato (*Lycopersicon esculentum* Mill), which belongs to the Solanaceae family (Pritesh et al. 2011). Tomato is typically produced in open fields; it is a high-value nutritional plant in both rural and urban regions (Maurya et al. 2019). Fruits can be juiced, dried, boiled, utilized fresh, or processed into sauce, ketchup, or other products. They are high in lycopene, vitamins A and C, modulate the risk of heart disease, breast and prostate cancers and other age-related disorders (Babalola and Glick 2012). Due to the economic and nutritional importance of tomato, their production has increased recently in Egypt. According to Agriculture and New Reclamation Lands report, the area planted with tomato reached 356,896 Fed in 2020 with 17.902 Tons/Fed average productivity and 6,389,295 Tons total productivity. Egypt exports tomato to several countries, biggest importers are Saudi Arabia Kingdom, Libya, Russian Federation, and United Arab Emirates (ITC calculated based on UN COMTRADE 2021).

Unfortunately, post-harvest diseases considerably reduce the production output in terms of both quality and quantity (Herrera-Téllez et al. 2019). Whereas, several fungal species, including *Fusarium* spp. have been shown to contribute to fruit rot. Such rot causes significant output loss, and it is frequently observed in markets (Baria et al. 2015; Edel-Hermann and Lecomte 2019). *Fusarium* spp. are phytopathogenic, soil-borne, toxin-producing, and have caused major health issues worldwide (Matos and Ricardo 2006). Tomato post-harvest losses in tropical areas range 20–50% during harvest, transportation, and consumption where crops are destroyed, and the yields are decreased (Agrios 2005; Pila et al. 2010). However, the development of alternative methods to treat diseases has been hampered by some problems, including the frequent use of chemicals, their side effects, the emergence of chemical resistance, the long-term survival of viruses, and most importantly the hazards to human health and the environment.

On one hand, the use of fungicides has proven to be quite efficient in limiting *Fusarium* growth and development. However, resistant isolates are created due to fungicides’ overuse, which also seriously endangers all living organisms (Baibakova et al. 2019; Taha et al. 2023). Therefore, alternative control methods are being widely investigated. Interestingly, the use of bioagents to prevent *Fusarium* wilt disease in tomato plants has been successful (Freeman et al. 2002; El-Hendawy et al. 2005).

On the other hand, one of the essential elements for humans is selenium (Se). It plays a vital role as co-enzymes and/or as antioxidant. Se element is an essential component for both human and animal, where > 25 human enzymes containing selenocysteine are necessary for human health (Papagiannopoulos and Sotiropoulos 2022). A typical adult requires 40–300 µg Se/day (Tran and Webster 2013). The use of SeNPs has fewer toxicological concerns as compared to bulk Se (Khurana et al. 2019). Importantly, NPs have been produced via eco-friendly techniques using microbial and plant sources (Elbaz et al. 2016; Zambonino et al. 2021; Elnady et al. 2022a, b). Green biosynthetic methods have been widely used to produce metallic NPs like gold, silver, zinc oxide, selenium, and others, because they are non-toxic, and compatible with many biomedical and food applications (Tayel et al. 2017; Elnady et al. 2022a, b). In addition, SeNPs possess additional bioactivities over bulk metal. As a result, bio-mediated SeNPs have been employed in many bio-applications, such as antimicrobial, anticancer, antioxidants, immune-modulatory formulations, and cytokine inducers (Alsaggaf et al. 2020; Huerta-Madroñal et al. 2021; Taha et al. 2023). In addition, SeNPs have been used to treat several diseases, including cancer, liver fibrosis, diabetes, inflammatory disorders, and drug-induced toxicity (Khurana et al. 2019). SeNPs also exhibit antifungal activity against plant diseases and was used in agriculture and in plant nutrition (Bano et al. 2021; Salem et al. 2022a, b). Accordingly, the present study aims to use bio-mediated SeNPs as potential antifungal agent against *Fusarium* spp. in vitro and for the first-time to control *Fusarium* infections in tomato fruits. The shelf-life and quality of treated tomato fruits were also investigated through some physico-chemical studies.

## Materials and methods

### Materials

Fenugreek *(Trigonella foenum-graecum)* seeds were obtained from the Medicinal and Ornamental plants Department, Horticultural Research Institute, Agriculture Research Center, Egypt. Cherry tomato *(Solanum lycopersicum var. cerasiforme)* were purchased from Perfect licensed company farm in Sadat City. Selenium salt (Na_2_SeO_3_) was obtained Sigma/Aldrich. Cultural components, such as, Peptone, Glucose, Yeast, and Malt extracts were purchased from Lobal Chemie, India. Cell culture materials used for cytotoxicity assessment were obtained from Cambrex BioScience (Copenhagen, Denmark), and the chemicals were purchased from Sigma/Aldrich, USA. Sterilized double distilled water was used throughout the experimental work.

### Cultivation of fungi

*F. oxysporum* NRRL 32931 and *F. moniliforme* NRRL 13616 were provided by Plant Diseases Research Institute, Agricultural Research Center, Egypt. Fungal spores were cultured on potato dextrose agar (PDA) medium. The medium was prepared as follows (g/L): 200 Potatoes, 20 Dextrose, and 15 Agar with final pH of 5.6 ± 0.2. Inoculated plates were incubated at 28 °C for 3–5 days. Developed fungi were stored at 4 °C and were maintained by periodic sub-culturing on PDA every month.

### Fenugreek seeds extraction and biosynthesis of SeNPs

20 g air-dried Fenugreek seeds powder was added to 100 mL distilled water, shaken in an orbital incubator at 150 rpm for 48 h at room temperature, followed by filtering using Whatman No. 1 filter paper. The homogenate was subjected to 10,000 rpm centrifugation, then the supernatant was removed and passed through a 0.24 µm sterile syringe filter. For the synthesis of SeNPs, 10 mM Na_2_SeO_3_ solution was prepared, combined with Fenugreek extract to obtain (1:1 v:v) concentration (Ramamurthy et al. 2013), the pH was adjusted at 6.5–7.0, and the final solution was incubated for 48 h at 150 rpm in orbital shaker (New Brunswick, CA).

### Characterization of bio-mediated SeNPs

#### UV–VIS spectrophotometry and X-ray diffraction (XRD)

During the biosynthesis, SeNPs formation was monitored and scanned between 300 and 700 nm using Shimadzu-T80 spectrophotometer (China), by sampling two mL of the produced SeNPs solution in a quartz cuvette. Using XRD through Rigaku Ultima IV X-ray diffractometer (Rigaku, Japan) operating at 30 kV/10 mA with CuK radiation (1.54060) over a scanning range (10° to 70°) of Bragg angles (2), the crystalline form of NPs were identified (Al-Qaraleh et al. 2022).

#### TEM, EDX, and FTIR analyses

The size and morphology of the biosynthesized SeNPs were examined using transmission electron microscope (FETEM, JSM-2100F, JEOL Inc.) at the Petroleum Research Institute in Cairo, Egypt. The sample was prepared and allowed to dry on a copper grid coated in carbon. TEM (Phillips EM 208S) operating at 120 kV voltage scanned the grid (Anu et al. 2017). A scanning electron microscope (SEM) equipped with an energy dispersive X-ray (EDX) analyzer (JSM-7600F, JEOL, Japan) operating at 20 kV was used for elemental analysis of NPs. Translucent sample discs were created for Fourier Transform Infra-Red (FT-IR) analysis (Bruker IR Affinity, Japan) with a resolution of 1 cm^−1^ and a wavelength range of 500 to 5000 cm^−1^ (Sheikhlou et al. 2020).

#### Antifungal activity of biosynthesized SeNPs

The antifungal activity of SeNPs was determined using disc diffusion assay. The pathogen was grown on PDA for 5–7 days at 28 °C and was used to obtain 1×${10}^{6}$ spores/mL spore suspension that were added to PDA medium in petri-dishes. 50 mg/mL stock of SeNPs was prepared in DMSO. Sterile filter paper discs (Hi media) were loaded with 40 µL of each SeNPs concentration in the range (0.39–25 mg/mL) that were chosen after preliminary experiment. 150 µL of fungal spore suspensions (1 × 10^6^ spores/mL) were added to 20 mL PDA medium after 2 days of incubation at 28 °C. The inhibition zones diameters were measured in mm after 2–4 days incubation at 28 °C (Salem et al. 2022a). Minimum inhibitory concentration (MIC) was identified visually after 48 h of incubation at 28 °C and was outlined as the concentration of SeNPs that completely inhibit fungal growth after 2–4 days. 50 mg of SeNPs were dissolved in 1 mL DMSO as stock solution, and the resulting solution were serially diluted in the range (0.39–0.17 mg/mL) and (3.125–1.3 mg/mL) for *F. oxysporum* and *F. moniliform,* respectively. 150 µL of fungal spore suspension (1 × 10^6^ spores/mL) were added to the culture medium and incubated for 48 h at 28 °C. For minimum fungicidal concentration (MFC), 1 mL of each concentration was added to 20 mL sterile PDA, poured into sterile petri-dishes and incubated at 28 °C for two days.

#### Application of SeNPs as biocontrol for post-harvest *Fusarium* infection of tomato

Cherry tomato fruits (*Solanum lycopersicum* var. *cerasiforme)* were obtained at three harvested stages (Groups A, B, C). Group A: Red colored, Group B: Yellow colored, and Group C: Green colored. The fruit’s average size ranged from 15 to 20 mm in diameter and weighed 7–10 g for each fruit, slightly flattened to oval and none of them had any visible infections or surface abrasions. Fruits were surface sterilized by immersion in sodium hypochlorite solution (1%) for 15 min followed by distilled water rinse and air drying.

Each group includes negative control (**T1)** that was treated with sterile water only, and (**T2**) was treated with SeNPs only. The third group (**T3**) was treated with SeNPs at (MIC, 2MIC, 3MIC) before their infection with the pathogen spores, and the fourth group (**T4**) was infected without treatment. Tomato fruits were submerged for 15 min in SeNPs solution at (MIC, 2MIC, 3MIC) then air-dried (Salem et al. 2022a, b). Tomato injury was done using a sterile needle (0.5 mm in diameter and 2 mm in depth), fruits were punctured manually three times along the equator. The artificial infections were performed using *F. oxysporum* spores’ suspension (1 × 10^6^ spores/mL) from 5 to 7 days old pathogen. The method for inoculating the fruits involved spraying the spore suspension in a volume of 1 mL onto each fruit and then air-dried. Each group consists of 30 fruits with three replicates. The treatments were stored at 5 °C and 25 °C with 92–95% relative humidity for 35 days. Development of the fungi on tomato fruits was monitored daily.

#### Microscopic observation of SeNPs-treated fungi

The morphology of *F. oxysporum* mycelial, after treatment with MIC of SeNPs was detected microscopically using digital optical microscope (Leica DMi8 S-platform, Germany), after incubation of fungi with SeNPs for 18 h under stirring, then staining the treated mycelia.

### Chemical analysis of tomato fruits

#### Titratable acidity (TA) and pH measurements

Tomato juice was extracted by homogenizing four tomatoes from each group for 1 min at high-speed using a food blender (Model: LM2201, Moulinex, China) and filtered using muslin cloth. The TA% of the tomato was determined by the titration method of Teka (2013). Two mL of the tomato juice was diluted with 38 mL of distilled water and phenolphthalein as an indicator. TA % of tomato juice was calculated by titrating two mL of tomato juice against 0.1 N NaOH. The TA % was calculated using the following equation (Eq. 1):

1 $$\mathrm{\% Citric\, acid }=\frac{\mathrm{vol}:\mathrm{NaOH }\left(\mathrm{mL}\right)\times 0.1 (\mathrm{normality\, of\, NaOH})\times 0.064}{(mL \,or g\, Juice) }\times 100$$

where 0.064 is the citric acid milliequivalent factor. 10 mL of tomato juice was poured into 50 mL beaker and the pH was measured using the pH-meter (Model: EUTECH Cyberscan, China).

#### Total lycopene and carotenoids

Total lycopene and carotenoids pigments were determined spectrophotometry based on Munhuewyi (2012) method. One gram of blended tomatoes was extracted by grinding in 14 mL hexane and acetone (3:2 v/v) solution using centrifuge (Model: Sanyo MSE Harrier 18/80, Sanyo, Tokyo, Japan) at 10,000 rpm for 10 min at 4 °C. The obtained supernatants were collected and completed to 25 mL. The absorbance was determined at 502 nm using UV/VIS spectrophotometer (Model: Lambd900, PERKIN ELMER, US). The following equations (Eqs. 2 and 3) were used for calculations.

2 $$\mathrm{Total\, Carotenoids }(\mathrm{\mu g}/\mathrm{g})=\frac{{OD}_{502}\times 410}{\mathrm{mass\, of\, the\, sample }(\mathrm{g})}\times 1000$$

3 $$\mathrm{Total\, Lycopene }(\mathrm{\mu g}/\mathrm{g})=\frac{{OD}_{502}\times 3.12}{\mathrm{mass\, of\, the\, sample }(\mathrm{g})}\times 1000$$

Lycopene and carotenoids were presented as mg.100 g^−1^ of fresh weight (Al-Dairi et al. 2021a).

### Physical quality analyses

#### The percentage of physiological weight loss (PWL)

PWL was determined according to the method described by Moneruzzaman et al. (2009). Tomato fruits within each treatment were weighed initially and after the storage period using digital balance. The weight loss was calculated for each interval day and was converted into percentage (Eq. 4) as:

4 $$\mathrm{PWL} \%=\frac{\mathrm{intial\, weight}-\mathrm{final\, weight}}{\mathrm{intial\, weight}}\times 100$$

where, initial weight was the weight taken before any treatment and final weight was the weight of fruits after fungal growth (Tolasa et al. 2021).

#### Percentage of fruit decay (PFD)

PFD was determined by counting the number of rotten tomato fruits divided by their total no. and expressed in percentage (Eq. 5) (Tolasa et al. 2021).

5 $$\mathrm{PFD} \mathrm{\%}=\frac{\mathrm{No}.\mathrm{ of\, decayed\, fruits}}{\mathrm{Total\, no}.\mathrm{ of\, fruits}}\times 100$$

#### Shelf life of tomato fruits

The shelf life of fruits was determined by calculating the number of days during fruits’ storage on shelf up to the stage it is still acceptable for marketing (Sinha et al. 2019). It was determined based on the appearance and spoilage of fruits. When 50% of fruits showed symptoms of shrinkage or spoilage, the sample was considered to have reached the end of shelf life (Tolasa et al. 2021).

#### Cytotoxicity assessment using Sulforhodamine-B protein (SRB)

SRB was used to investigate the cytotoxicity of bio-mediated SeNPs on healthy Human Skin Fibroblast (HSF) and Oral Epithelial Cells (OEC) cell lines at Nawah Scientific Inc. (Mokatam, Cairo, Egypt). Cells were kept alive in Dulbecco's Minimum Essential Medium (DMEM), humidified CO_2_ (5% v/v), 10% heat-inactivated foetal bovine serum, streptomycin (100 mg/mL), penicillin (100 U/mL), and 37 °C. 96-well plates were filled with 100 µL aliquots of cell suspension (5 × 10^3^ cells), which were then cultured in DMEM for 24 h. A second portion of 100 mL of DMEM with different SeNPs concentrations was used for cells treatment. After 72 h of exposure, treated cells were fixed by removing the medium and replacing it with 150 µL of 10% Trichloroacetic acid (TCA), which was then incubated for 1 h at 4 °C. After the TCA solution was withdrawn, distilled water was used to wash the cells five times. At room temperature, aliquots of a 70 µL SRB solution (0.4% w/v) were added and incubated for 10 min in the dark. Acetic acid (1%) was used to wash the plates, then left to air dry overnight. The protein-bound SRB stain was then dissolved in 150 µL of TRIS (10 mM), and the absorbance was determined at 540 nm using a BMGLABTECH®-FLUO star Omega microplate reader (Ortenberg, Germany). The following equation was used to compute relative viability (%), and the IC_50_ was derived (Eq. 6) from the dosage response curve.

6 $$\mathrm{ Absorbance\, of\, treated\, cells}/\mathrm{Absorbance\, of\, control\, cells})] \times 100$$

#### Detection of selenium metal in treated tomato fruits using ICP-MS

Metal concentration was measured using inductively coupled plasma-mass spectroscopy (ICP-MS) (iCAP, Thermo, Germany) at accredited food and soil laboratory located at GEBRI, University of Sadat City, Egypt. The laboratory has EGAC/ILAC accreditation number 217006. The amounts of Se metal in treated tomato fruits were determined and compared with untreated ones. The studies included certified reference materials (Merck, Germany). The average and relative standard deviation were calculated using Qtegra program (APHA 2017).

### Statistical analysis

Results were expressed as the means ± standard deviation. Data were analyzed by Two-way ANOVA to find significant differences using MSTAT-C (MSTAT Development Team). The least significant difference (LSD) among levels of each treatment was compared using LSD test at 5% level according to Steel et al. (1997).

## Results

### Bio-mediated SeNPs synthesis and its characterization

**Fig. 1:**
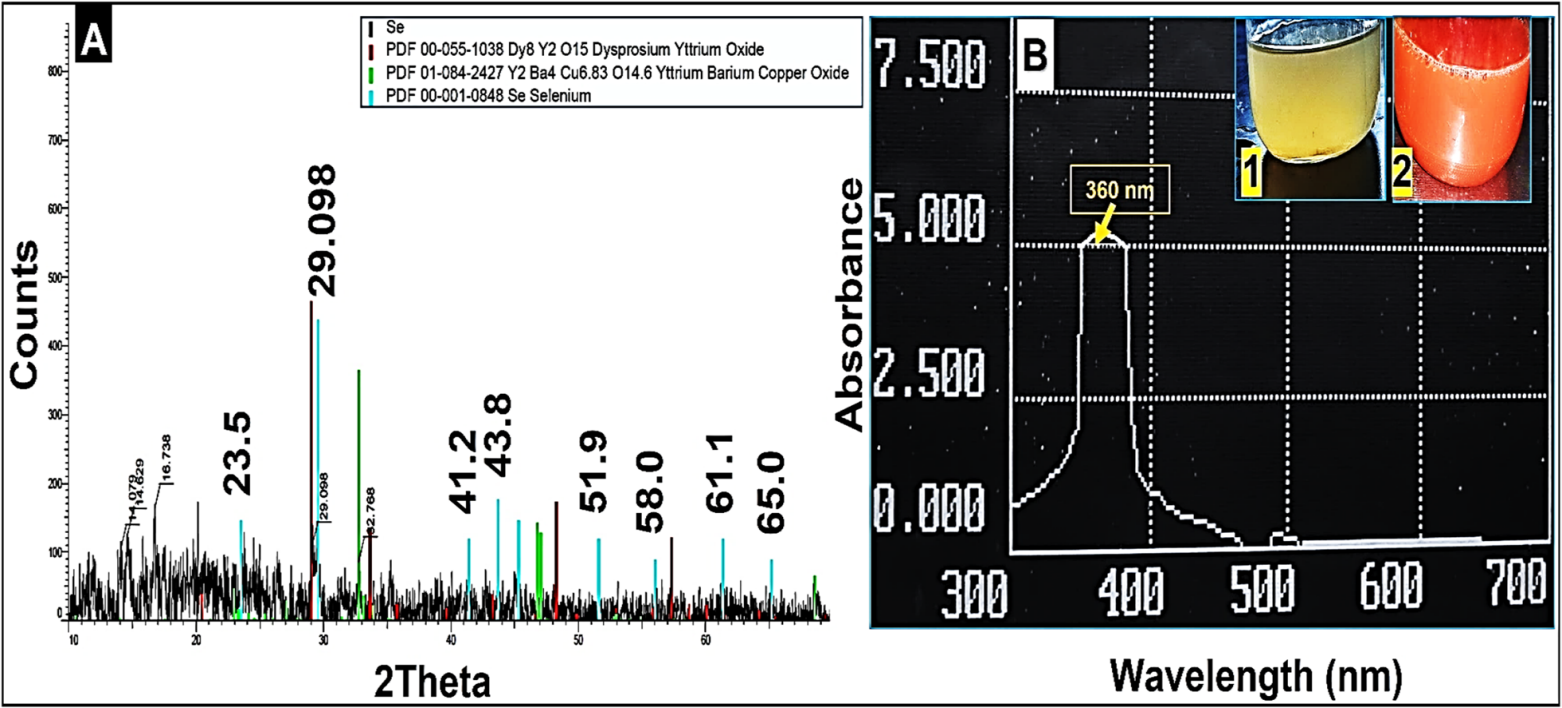
XRD analysis (**A**) and UV–VIS spectroscopy (**B**) of SeNPs biosynthesized using Fenugreek seeds aqueous extract, 1 is the aqueous extract and 2 is the reaction color after SeNPs formation

**Fig. 2:**
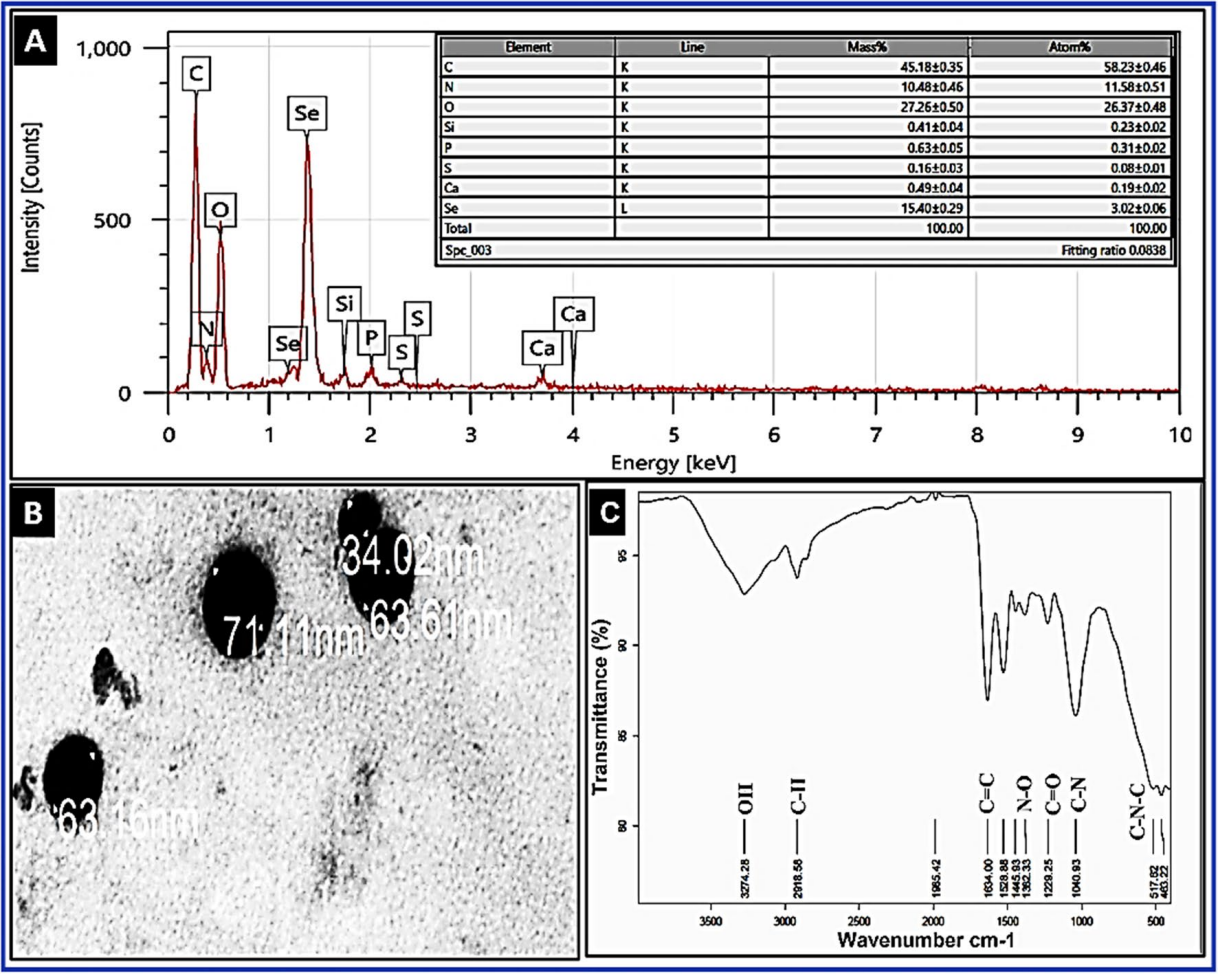
EDX (**A**), TEM micrographs (**B**), and FTIR analysis showing different functional groups acting as bio-reductant and stabilizing agent (**C**) for SeNPs biosynthesized using Fenugreek seeds aqueous extract

SeNPs were successfully produced extracellularly within 48 h of incubation as confirmed by the brick red color detected using UV–VIS spectroscopy by a sharp absorption band at 360 nm and XRD analysis (Fig. 1A and B). The XRD design the number of Bragg reflections with 2ϴ◦values of (23.5°, 29.09°, 41.2°, 43.8°, 51.9°, 58.0°, 61.1, 65.0°) by the biosynthesized SeNPs corresponds to crystal planes (100), (101), (110), (102), (201), (112), (003), and (210), respectively (Fig. 1A). These diffraction peaks were confirmed with Joint Committee on Powder Diffraction Standards (JCPDS) file no. 06-0362. EDX analysis revealed three distinct signals, a strong signal from the Se atom (15.4%), as well as signals from the O and C atoms (27.26 and 45.18%), respectively (Fig. 2A). The elemental composition and the purity of the SeNPs was determined using EDX, and other elements, such as N, Si, P, S, were detected with less pronounced peaks. The morphology of SeNPs was detected by TEM to be spherical, uniformly distributed, with average size between 34.02 and 63.61 nm (Fig. 2B). FTIR analysis demonstrated several absorption peaks corresponding to different biomolecules present in the biological extract used during biosynthesis (Fig. 2C).

### Antifungal activity of biosynthesized SeNPs

The aqueous extract of Fenugreek seed had no effect on *Fusarium* spp. However, the biosynthesized SeNPs had a high antifungal effect giving inhibition zones of 31 mm and 27 mm against *F. oxysporum* and *F. moniliforme*, respectively (Table 1). Biosynthesized SeNPs exhibited good antifungal activity against the two fungal strains (*F. oxysporum* and F. *moniliforme)* with concentration ranging from 0.17–0.39 mg/mL and 1.4–3.125 mg/mL, respectively. The MICs of SeNPs were 0.25 and 1.7 mg/mL*,* and the MFCs were 0.27 and 2.9 mg/mL for *F. oxysporum* and *F. moniliforme,* respectively (Table 1).

**Table 1:** Antifungal activity of SeNPs against two *Fusarium* spp. expressed as mean inhibition zone diameter (ZOI) of three replicates in mm ± SD, MIC and MFC were calculated as mg/mL, inhibition zone of *F. moniliforme* (**A**) and *F. oxysporum* (**B**) using different concentrations of SeNPs (mg/mL)

Sample	*Fusarium oxysporum*			*Fusarium moniliforme*		
	ZOI (mm)	MIC (mg/mL)	MFC (mg/mL)	ZOI (mm)	MIC (mg/mL)	MFC (mg/mL)
Fenugreek aqueous extract	0	ND	ND	0	ND	ND
Bio-mediated SeNPs	31 ± 0.14	0.25	0.27	27 ± 0	1.7	2.9
	

### Effect of SeNPs coating on *F. oxysporum*-induced post-harvest disease

**Fig. 3:**
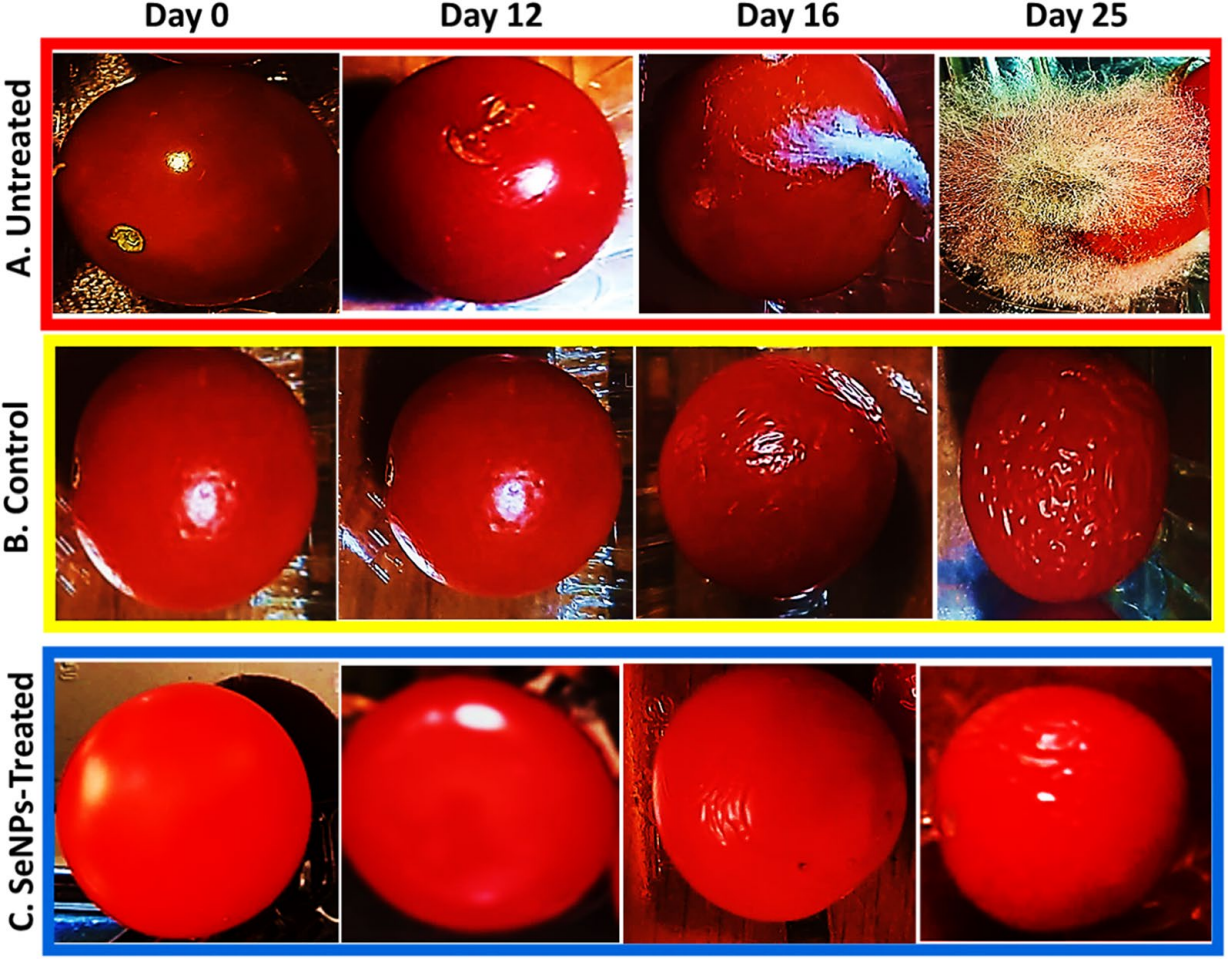
Infected tomato fruits after nano treatment, **A**: untreated; **B**: water-dipped (control); **C**: treated with SeNPs at MIC after 25 days of storage

The treatment of red colored tomato fruits (Group A) revealed a significant difference between SeNPs-treated infected fruits (**T3**) and untreated infected fruits (**T4**) (Fig. 3). After 16 days of infection with *F. oxysporum*, tomato fruits treated with SeNPs had no infection signs. While the control fruits (untreated) were decayed with the appearance of fungal growth, the treated fruits exhibited 100% reduction of infection. Interestingly, the fruits’ coating with SeNPs could maintain the quality of treated fruits for further 20 days without any infection signs.

Results (Fig. 3) demonstrated the efficiency of SeNPs in protecting tomato fruits from *F. oxysporum* infection in vivo. The coating with SeNPs completely protected fruits from any infection signs (100% reduction) and preserved the fresh-like appearance of treated fruits when stored at 25 °C and 5 °C for 25 days and 35 days, respectively. This effect was observed when compared with the untreated infected fruits (**T4**) that exhibited 60% and 20% infection when stored at 25 °C and 5 °C, respectively. Tomato fruits infected with *F. oxysporum* showed pale white powdery lesions on the fruit's surface, covered with white/pinkish fungal mycelium (Fig. 3). Comparing the infected area to other healthy parts, a soft slightly sunken, and decayed appearance was detected. The treatment with SeNPs at its MIC of 0.25 mg/mL successfully inhibited *F. oxysporum* growth in vitro*,* with 100% protection in treated tomato fruits up to 25 and 35 days when fruits were stored at 25 °C and 5 °C, respectively.

**Fig. 4:**
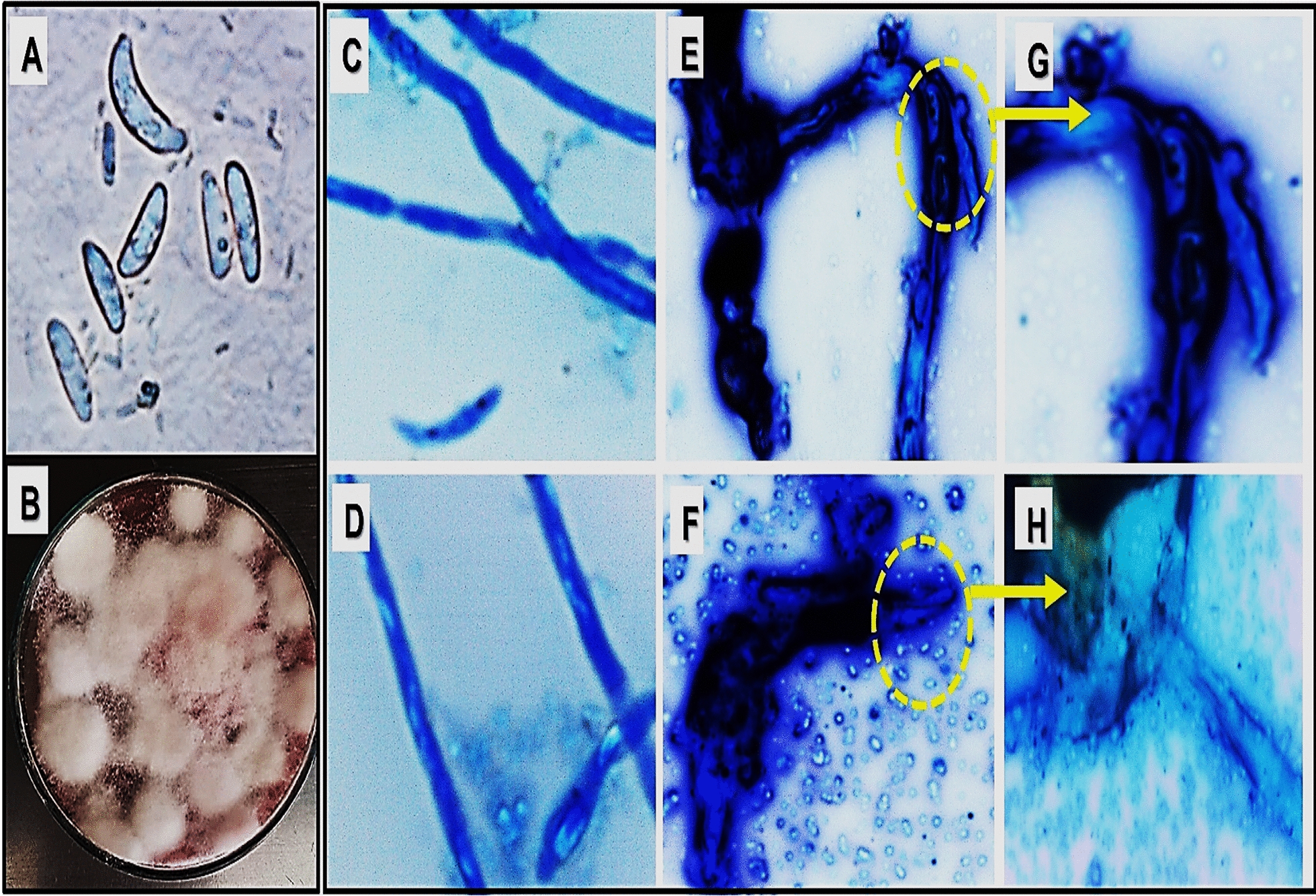
Microscopic examination of *Fusarium oxysporum* (*400) showing its charcteristic spores (**A**), macroscopic examination on potato dextrose agar medium (**B**), untreated fungul mycelium control (**C**, **D**), Treated fungal mycelium with MIC of SeNPS showing lysis and complete destruction after 18h of treatment (**E**–**H**)

**Fig. 5:**
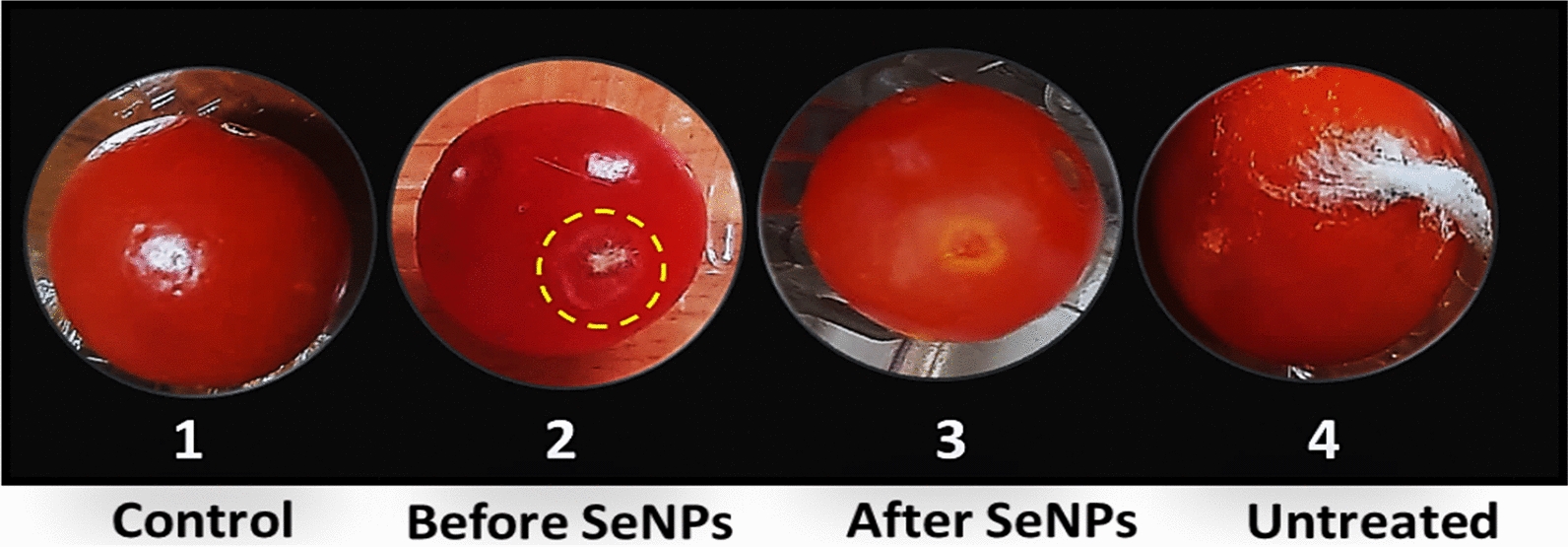
Effect of SeNPs treatment after infection with *Fusarium oxysporum*, 2: shows the beginning of infection, 3: shows the treatment effect on disease progression as compared with 4 representing untreated infected fruits

The alteration in *F. oxysporum* mycelial morphology after the treatment with MIC of SeNPs was microscopically detected (Fig. 4). The mycelium of *F. oxysporum* appeared healthy and uniform before SeNPs treatment (Fig. 4A–D). After 18 h of SeNPs treatment, the growth of fungi was limited, and its mycelium showed irregular swellings and fragmentations, with the appearance of high distortion signs (Fig. 4E–H). Interestingly, SeNPs was applied to infected tomato fruits to assess its potential to lower the pathogen infection rate (Fig. 5). The fungal growth was clearly visible before the fruits were treated by SeNPs at its MIC as compared to the untreated infected fruits, however the treatment restricted the growth and prevented further disease progression.

### Effect of storage temperature on disease progress and shelf-life of tomato fruits

For red colored fruits (Group A), its appearance, color, and overall quality of treated fruits (**T3**) were maintained throughout the storage duration for 25 and 35 days at 25 °C and 5 °C, respectively, with no fungal infection or symptoms (Table 2). However, changing the temperature from 5 to 25 °C affected the fruits’ quality. For **T1** group, tomato sprayed with water only kept their fresh appearance for 6 days only***,*** then started to shrink and lose their shape. While non-infected fruits treated with SeNPs (**T2**) kept their good appearance for 12 days. Moreover, the variations of storage temperature have greatly impacted the pathogen growth, where no growth was seen on infected fruits during their storage at 5 °C for 35 days, but after changing the temperature to 25 °C, the fungal growth was visualized on infected untreated group (**T4**) after 5 days, with 50% infection that increased to reach 100% after 6 more days. This is because fruits that have been stored at 5 °C are less likely to develop fungi, providing non-optimum temperature for fungal growth being 25 °C or higher. However, the fungal infection was reduced by 80% for infected fruits treated with MIC of SeNPs (**T3**) after 10 days.

**Table 2:** Effect of temperature change on diseases progress and shelf-life of tomato fruits

Temperature (25 °C)	Red colored group (A)				Yellow colored group (B)			
	Water only (T1)	SeNPs only (T2)	Infected treated (T3)	Infected untreated (T4)	Water only (T1)	SeNPs only (T2)	Infected treated (T3)	Infected untreated (T4)
6 days	Fresh +	Fresh + +	No Infection	50% PFD	Fresh + +	Fresh + + +	No Infection	ND
10 days	Shrinkage +	Fresh +	20% PFD	100% PFD	Fresh +	Fresh + +	No Infection	ND
12 days	Shrinkage + +	Fresh +	20% PFD	–	Shrinkage +	Fresh +	No Infection	66% PFD
15 days	Shrinkage + + +	Shrinkage +	20% PFD	–	Shrinkage + +	Fresh +	No Infection	70% PFD

Data are average of three replicates for each treatment, each replicate contains 10 fruits. Percentage of fruit decay (PFD) was determined by counting the number of tomato fruits showing infection periodically divided by total no of fruits *100 (Tolasa et al. 2021). Infected Treated fruits were treated with MIC value of SeNPs. Shrinkage or freshness were taken on scale, +  +  + : high, +  + : moderate and + : low

For yellow colored tomato fruits (Group B), no fungal growth was observed on all fruits during their storage at 5 °C for 35 days, but the fungal growth was observed after 12 days on infected untreated fruits (**T4**) only, exhibiting 66% infection after the change of temperature to 25 °C. However, tomato fruits sprayed with water only (**T1)** kept their fresh appearance for 10 days after the temperature’s change, then started to shrink and lose their shape, while non-infected fruits treated with SeNPs (**T2**) kept their good appearance for 15 days. However, infected fruits treated with MIC of SeNPs (**T3**) had 100% disease reduction (Table 2). For green colored fruits (Group C), no change was observed for all treatments during the whole experiment. Also, non-infected tomato fruits sprayed with either water or SeNPs only (**T1** and **T2**), as well as non-injured fruits for all groups didn’t show any pathogen growth or disease symptoms.

### Cytotoxicity assessment of SeNPs

**Fig. 6:**
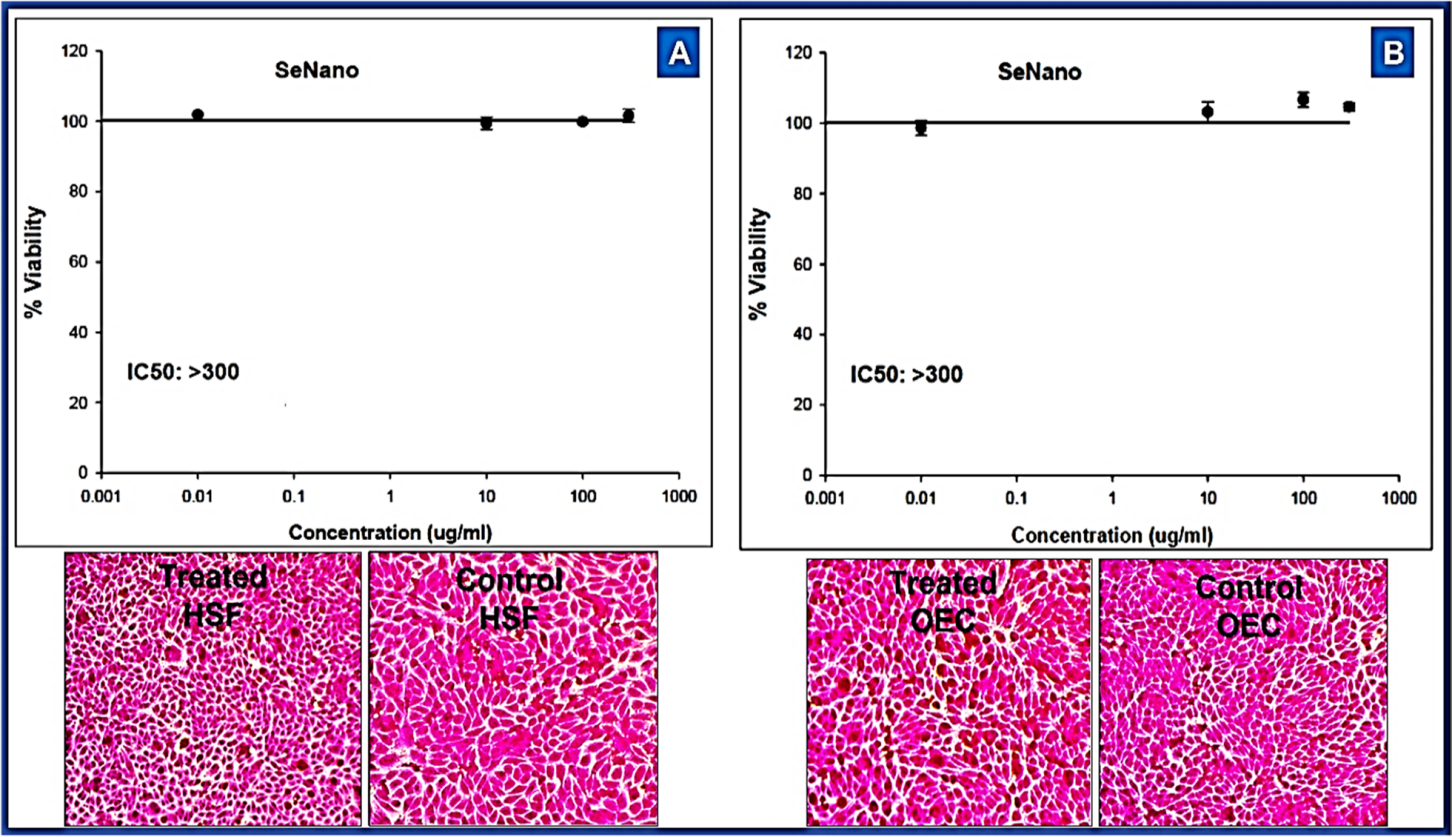
Cytotoxic effect of SeNPs biosynthesized using Fenugreek seeds aqueous extract against normal Human Skin Fibroblast (HSF) and Oral Epithelial cells (OEC) using SRB assay, data expressed as the mean value of cell viability (% of control) ± S.D and IC_50_ was calculated

The cytotoxicity of biosynthesized SeNPs were tested using SRB at concentration (0.03–300 µg/mL) on two normal cell lines. Bio-mediated SeNPs did not show any visible cytotoxicity against OEC and HSF cell lines with IC_50_ > 300 μg/mL. Results also show a mild diminution in the cell viability using SeNPs concentration of 300 μg/mL (Fig. 6). Biomediated SeNPs have no harmful effect on the cell viability (> 90%) of HSF and OEC cell lines at all tested concentrations.

ICP/MS detected 0.019 mg/mL of Se metal in treated tomato fruits, as compared with the untreated control (0.001 mg/mL), after 25 days of storage at 25 °C. ICP/MS analysis suggest that only 1/13 of the concentration used for treatment is still present in the tomato fruits after 25 days of treatment. This concentration is still beyond the IC_50_ (> 0.30 mg/mL) and enough to keep the fruits freshness with shelf-life storage up to 25 days at 25 °C. In addition, this concentration protected the fruit from *Fusarium* infection by 100% and restricted the fungal growth if infection occurs.

### Chemical and physical analysis of tomato fruits

#### Titratable acidity (TA%)

**Fig. 7:**
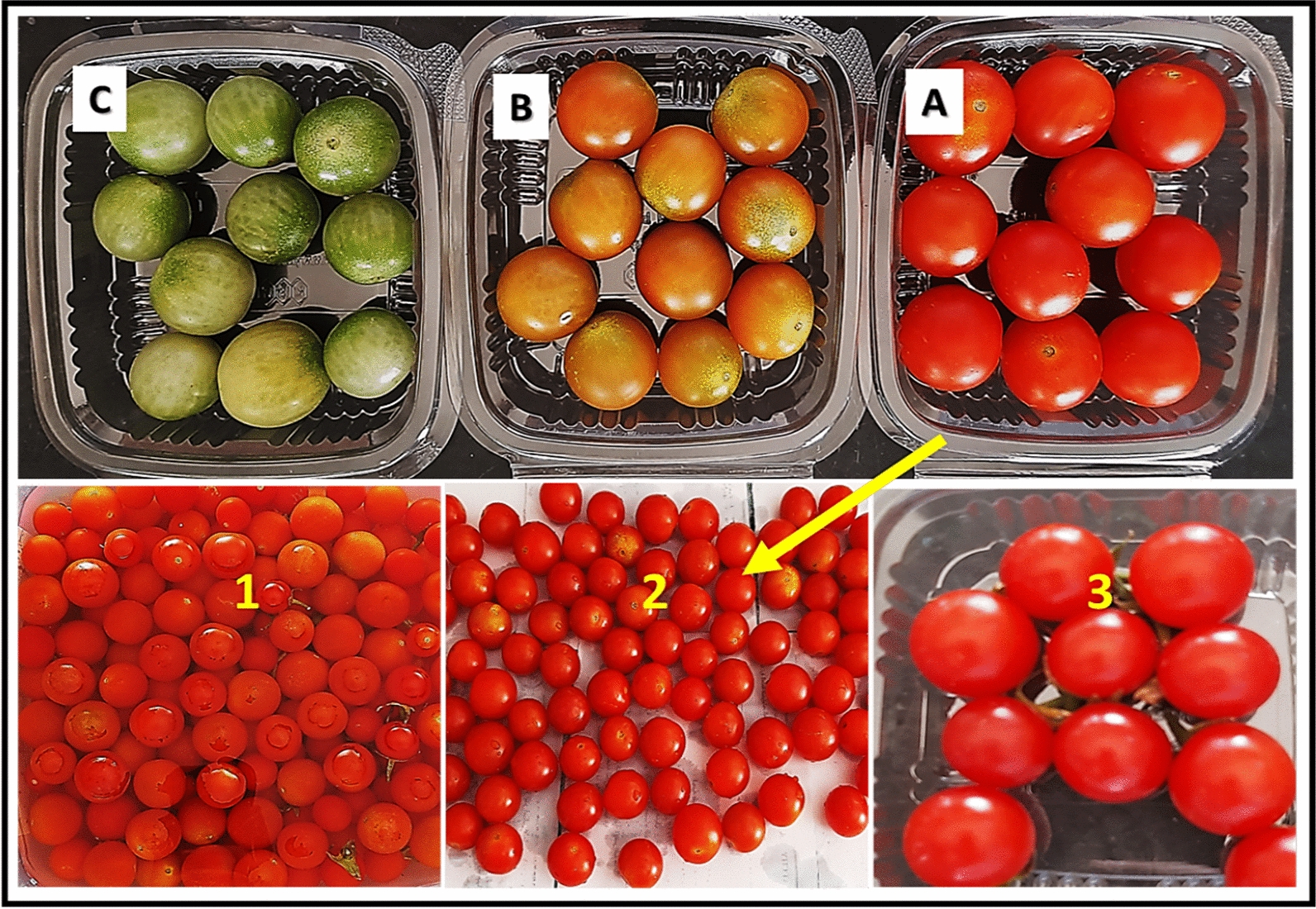
Preparation of the three groups of *Cherry* tomato showing different ripping stages, Group A (red colored), Group B (yellow colored), and Group C (green colored), Group A fruits during their 1: washing, 2: air-drying and 3: packaging

**Fig. 8:**
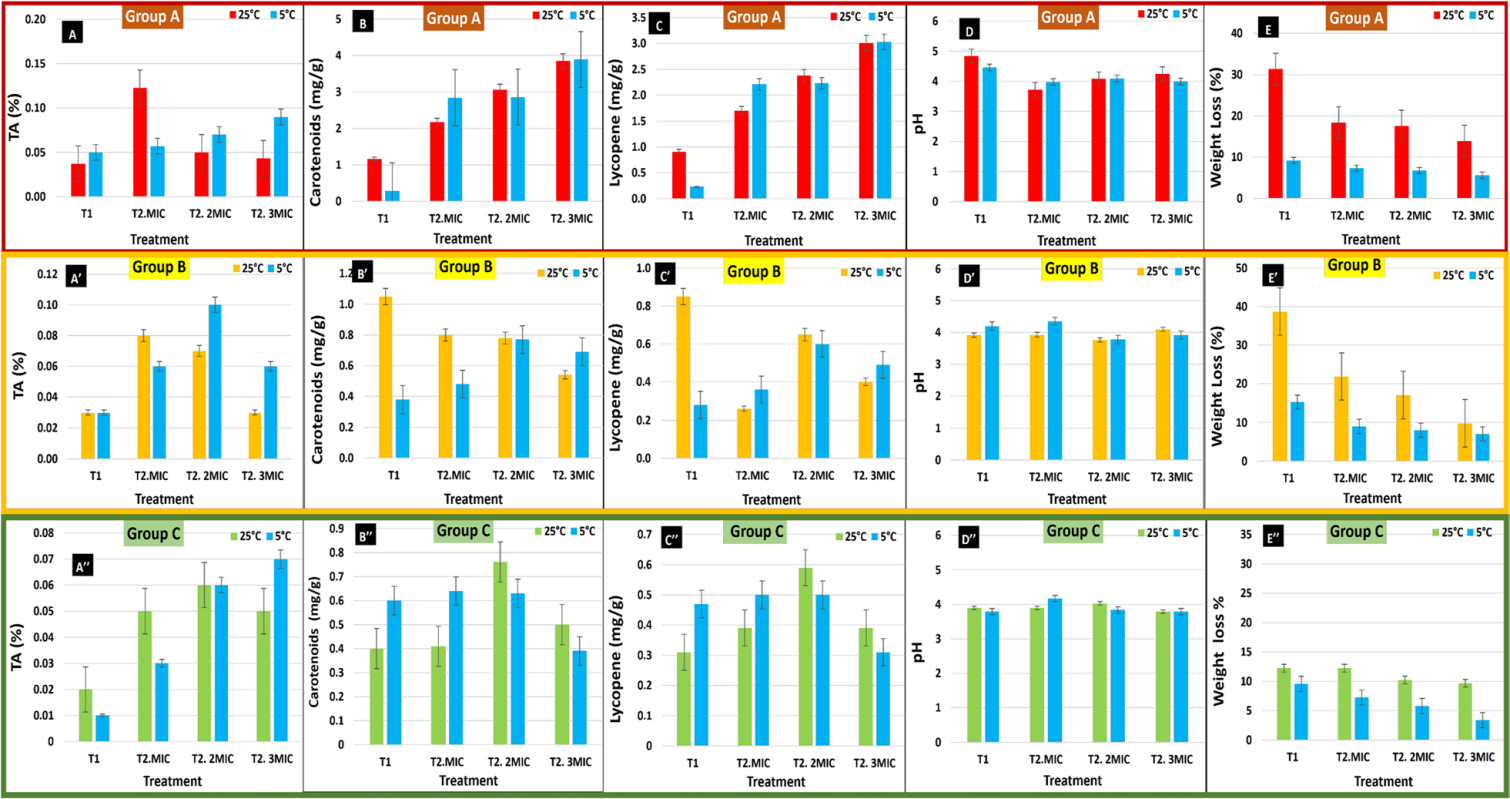
Effect of SeNPs-treatment of tomato fruits on some chemical and physical properties at two storage temperature (5 °C and 25 °C) for three ripening stages, Group A: Red colored (**A**, **B**, **C**, **D**, **E**), Group B: Yellow colored (**A’**, **B’**, **C’**, **D’**, **E’**), and Group C: Green colored (**A’’**, **B’’**, **C’’**, **D’’**, **E’’**). **T1**: negative control sprayed with water only, **T2**: non-infected treated fruits with SeNPs at MIC, 2MIC and 3MIC. *MIC* minimum inhibitory concentration for *Fusarium oxysporum* infection

The three groups of tomato fruits (A, B and C) (Fig. 7) were stored at 5 °C and 25 °C to assess the effect of temperature as well as SeNPs treatment on the physico-chemical properties as compared with negative control sprayed with water only (**T1**) (Fig. 8). At higher temperature (25 °C), the TA% reduction was accelerated as compared to 5 °C. However, treated fruits with SeNPs at MIC (**T2**) had significantly higher TA% as compared with the control (**T1**). Results revealed that SeNPs treatment of tomato fruits with MIC value increased the TA% as compared to untreated ones (**T1**) (Fig. 8A), which will probably increase the shelf life of treated tomato fruits. For Groups B and C, no significant difference in TA% was observed between the two storage temperatures (Fig. 8A, A’, A’’).

#### Total carotenoids and lycopene

Results obtained **(**Fig. 8B–B’’and C–C’’) revealed that total carotenoids and lycopene of red-colored tomato fruits (Group A) were significantly increased (2.51–3.87 mg/g) for fruits treated with SeNPs (**T2**) at different MIC values as compared with the control (**T1**) being 0.73 mg/g after 25 days of storage. However, there was no significant difference between those fruits stored at 5 °C or 25 °C. Also, yellow-colored fruits (Group B) have significant higher total carotenoids and lycopene for SeNPs-treated fruits (**T2**) as compared with control (**T1**) for those fruits stored at 25 °C, and significant difference between those stored at 25 °C and 5 °C being the highest at 25 °C. While for the green-colored tomato fruits (Group C), there was no significant difference between those fruits stored at 25 °C and 5 °C or those treated and the control (Fig. 8B’’and C’’).

#### pH change in tomato fruits

Results (Fig. 8D, D’, D’’) revealed that the pH value of red-colored fruits (Group A) treated with SeNPs (**T2**) (pH of 3.85) was significantly decreased as compared to control (**T1**) (pH 4.65) after 25 days of storage. However, there was no significant difference between those stored at 5 °C or 25 °C. But for yellow and green colored fruits (Groups B and C), there was no significant difference between treated fruits and control for all fruits. The decrease in pH values agrees with the TA% reduction that was decelerated by the SeNPs treatment (Fig. 8A–A’’).

#### Physiological weight loss percentage

Results revealed that weight loss % was significantly increased for all tomato fruits groups stored at 25 °C up to 2.8-folds than those stored at 5 °C. Results (Fig. 8E–E’’) also revealed that weight loss % of all tomato fruits groups (A, B, C) was significantly decreased for fruits treated with SeNPs (**T2**) at different MIC values as compared with the control (**T1**). Being in the range (9.75–12.89%) and (8.43–15.47%) for Group A and B, respectively, as compared with the control (20.30 and 26.98%). Remarkably, weight loss % was significantly decreased by increasing the MIC used for treatment (Fig. 8), where treated fruits with SeNPs maintained their appearance and color by increasing SeNPs concentration. The treatment of tomato with SeNPs (MIC, 2MIC, 3MIC) was effective in maintaining the weight of fruits during their storage at 25 °C and 5 °C for 25 and 35 days, respectively. Overall results (Fig. 8) indicate that post-harvest storage temperature as well as SeNPs treatment affect the characteristics of fresh tomato. However, the treatment of tomato fruits with SeNPs at its MIC value positively affected the chemical properties of tomato fruits including TA%, pH, total carotenoids and lycopene, as well as decreased the physiological weight loss %. This effect is probably due to the barrier effect of SeNPs acting against microbial growth and preserving freshness, thus increasing fruits’ shelf life up to 25 and 35 days when stored at 25 and 5 °C, respectively. Therefore, 5 °C was chosen as the storage temperature for the next experiment.

#### Chemical analysis of tomato fruits stored at 5 °C

**Fig. 9:**
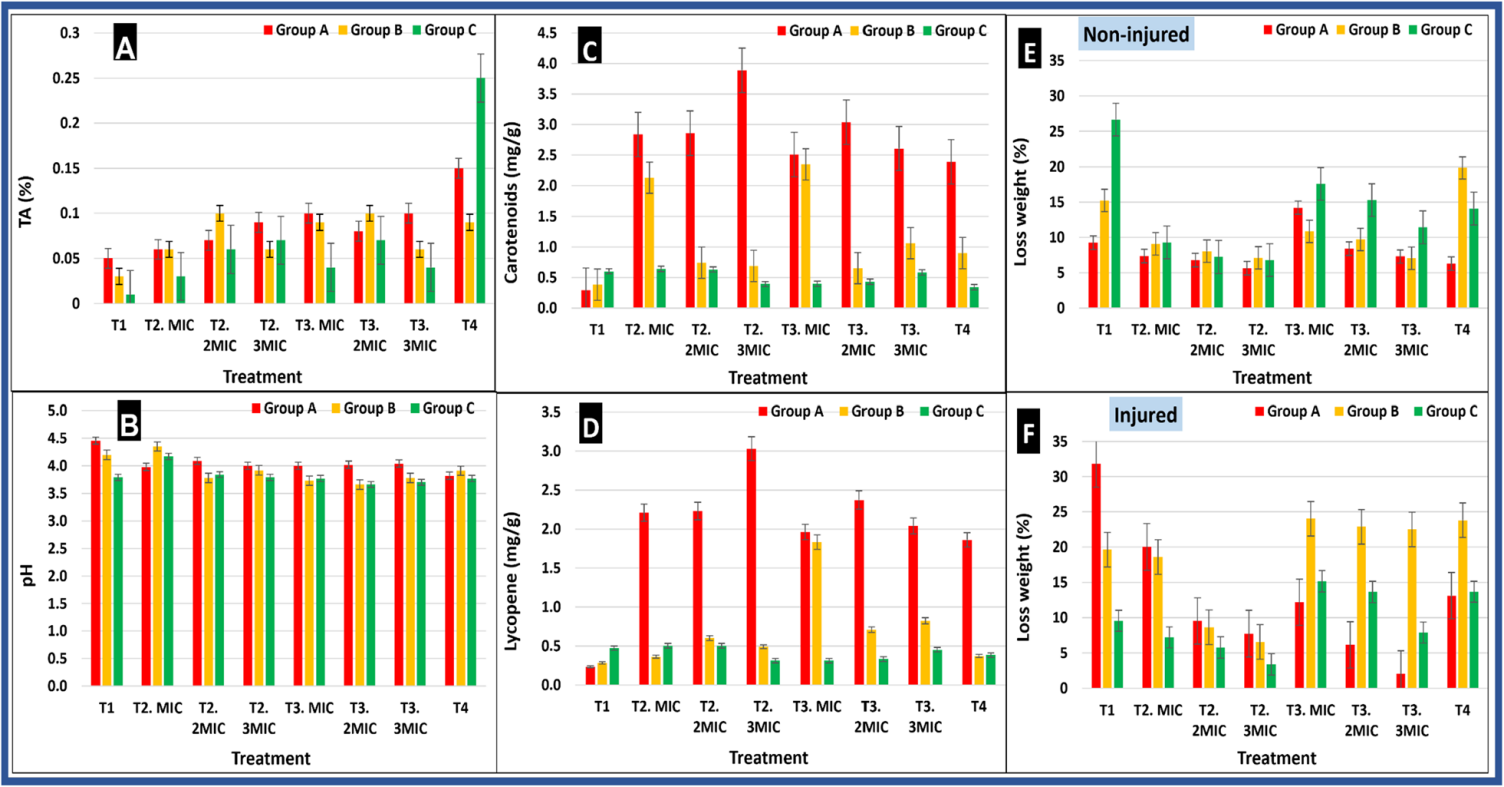
Effect of SeNPs-treatment of tomato fruits on some chemical (**A**–**D**) and physical (**E**, **F**) properties for three ripening stages at storage temperature (5 °C), Group A: Red colored, Group B: Yellow colored, and Group C: Green colored. **T1**: negative control sprayed with water only, **T2**: non-infected treated fruits with SeNPs at MIC, 2MIC and 3MIC, **T3**: infected treated fruits with SeNPs at MIC, 2MIC and 3MIC, **T4**: infected untreated fruits. *MIC* minimum inhibitory concentration for *Fusarium oxysporum* infection

Results (Fig. 9A) revealed that, all treated fruits in all groups showed insignificant difference in TA % as compared with each other or with the control (**T1**), however infected untreated fruits with SeNPs (**T4**) had significantly higher TA% as compared with the control (**T1**) and with all treated fruits (**T2** and **T3**). In addition, results (Fig. 9B) revealed that total carotenoids and lycopene of red-colored tomato fruits (Group A) was significantly increased in treated fruits with SeNPs (**T2** and **T3**) as well as infected untreated fruits (**T4)** being (2.39–3.89 mg/g) and (1.86–3.03 mg/g), respectively, as compared with untreated ones (**T1**) (0.23 and 0.29 mg/g). However, there was a significant difference between the three tomato groups, which is normal since they were showing different ripping stages with different content of carotenoids and lycopene. Also, the pH of all tomato fruits was lower than the negative control (**T1**) being the lowest (pH 3.82) for infected untreated fruits (**T4**) which agree with previously detected TA % (Fig. 9C) as evidence for the fungal infection. Finally, results (Fig. 9D) revealed that the weight loss % of all tomato fruits groups (A, B, and C) was significantly decreased for all fruits treated with SeNPs (**T2, T3**) at different MIC values as compared to control (**T1**) and to infected untreated ones (**T4**). As a result, the treatment with SeNPs (MIC, 2MIC, 3MIC) was effective in maintaining the fruits’ weight during their storage. Weight loss % was significantly decreased (2.04–12.20%) by SeNPs treatment especially for infected treated fruits (**T3**) being the lowest for those treated with 3MIC value as compared with infected untreated ones (**T4**) (13.3%).

## Discussion

### Bio-mediated SeNPs synthesis and its characterization

Searching for effective and safe alternatives to chemical fungicides is needed globally as chemicals have been associated with negative health impacts. In this respect, bio-mediated SeNPs using Fenugreek seeds aqueous extract was used for the first time as potential antifungal bioagent against two *Fusarium* spp. The current study describes the biosynthesis of SeNPs using Fenugreek seed aqueous extract as an ecofriendly method. Fenugreek seed extract had several naturally occurring bioactive compounds, such as alkaloids, flavonoids, phenols, amino acids, glycosides, and polysaccharides with reducing bioactivity (Ramamurthy et al. 2013). These biomolecules successfully bio-mediated the synthesis of SeNPs by reducing selenite salt to SeNPs. The low cytotoxic effect of biosynthesized SeNPs is probably attributed to the various functional biomolecules found in the bio-extract used for synthesis. The treatment of tomato fruits with SeNPs completely protected it from any infection signs (100% reduction) and preserved the fresh-like appearance and color when stored at 5 °C or 25 °C, indicating possible application of SeNPs at its MIC during storage, and transportation. SeNPs characterization was primarily done using UV–VIS, which detected the biosynthesized SeNPs at 360 nm with brick red color. Similarly, Al-Qaraleh et al. (2022) reported the color change of reaction mixture from colorless to brick red with maximum absorption between 260 and 350 nm. This was caused by Surface Plasmon Resonance of the formed NPs, confirming the bio-reduction of Na_2_SeO_3_ solution to Se^0^ element by *Moringa peregrine* aqueous extract. The XRD patterns verified seven intense peaks that correspond to the crystallographic planes of Se crystals, thus confirming the nano-crystalline nature of biosynthesized SeNPs as compared with standard file no. 06-0362 (Ingole et al. 2010). Similarly, SeNPs crystal structure and phase composition were identified by Srivastava and Mukhopadhyay (2015), where the reflections of pure Se crystal at 23.6°, 29.9°, 41.4°, 43.8°, 51.8°, 55.9°, 61.8°, 65.3°, and 68.3° were attributed to the Bragg reflection peaks at (100), (101), (110), (102), (111), (201), (003), (202), (210), and (211). The FTIR analysis demonstrated various absorption peaks corresponding to different biomolecules present in the biological extract used for biosynthesis. The bands at 2918.58 cm^−1^ are typical bands for polysaccharides as determined by C-H symmetric/asymmetric stretching (Salem et al. 2022a). The C=C alkene group was detected at 1634 cm^−1^ (Anu et al. 2017). The C–N stretching of amines was detected at 1040.93 cm^−1^ and the C–N–C bending bands at 517.62 cm^−1^ and 463.22 cm^−1^ (Alagesan and Venugopal 2019; Al-Qaraleh et al. 2022). Typical absorption peaks for OH stretching and C–H vibration were detected at 3274.28 cm^−1^ and 1445.93 cm^−1^, respectively (Mellinas et al. 2019). The N–O stretching group is responsible for the peaks at 1423.47 and 1382.33 cm^−1^. The carboxyl group (C=O) stretching vibration peaks was detected at 1229.25 cm^−1^ (Satgurunathan et al. 2017). Effective stabilizing and/or reducing agents in the bioextract are due to the existence of important functional groups, such as O–H, N–H, C–N, C–H, N–O, C–N–C, and C=O as previously proposed by Elnady et al. (2022a, b) that is probably responsible for the bio-reduction and stability of SeNPs.

### Antifungal activity of biosynthesized SeNPs and its mode of action

The antifungal potentialities of diverse nanometals such as, Se, Ag, Cu, and TiO_2_ have been confirmed against some phytopathogenic fungi such as, *P. digitatum, A. alternata,* and *Aspergillus* spp. (Ouda 2014; Sánchez-López et al. 2020).

In the current study, biosynthesized SeNPs using Fenugreek seeds extract showed high antifungal effect against *F. oxysporum* and *F. moniliforme* as detected by MIC and MFC. The MICs of SeNPs were 0.25 and 1.7 mg/mL, and the MFCs were 0.27 and 2.9 mg/mL againt *F. oxysporum* and *F. moniliforme*, respectively. These values were much lower than those determined by Salem et al. (2022a) who reported that the composite SeNPs/pomegranate peel extract revealed MFC that ranged 22.5–25 mg/mL against *Penicillium digitatum*.

In addition, El-Saadony et al. (2021), reported that biosynthesized SeNPs using *Lactobacillus acidophilus* inhibited some *Fusarium* spp. in the range of 20–40 µg/mL. While, wheat supplemented with 100 µg/mL of SeNPs significantly reduced the incidence of crown-root rot disease in wheat by 75% and improved its growth, grain quality and quantity by 5–40%. Furthermore, Joshi et al. (2021) reported that infected tomato plants coated with biosynthesized SeNPs exhibited a significant protection (72.9%) against late blight disease caused by *Phytopthora infestans*.

The biosynthesized SeNPs interact with the microbial cell wall leading to disruption and alteration in its permeability, NPs enter the cell and inhibit the proteins and DNA synthesis. The antimicrobial activity of SeNPs is probably attributed to reactive oxygen species (ROS), such as hydroxyl radicals, superoxide anions, and hydrogen peroxides. ROS induce damage to the microbial cell membrane, inhibiting the DNA replication and amino acid synthesis (Filipović et al. 2021; Elnady et al. 2022a, b).

### Effect of SeNPs coating on *F. oxysporum*-induced post-harvest disease

The treatment with SeNPs at its MIC (0.25 mg/mL) successfully inhibited *F. oxysporum* growth in vitro*,* with 100% protection of treated tomato fruits up to 25 and 35 days when stored at 25 °C and 5 °C, respectively. The treatment of red colored tomato fruits (Group A) with biomediated SeNPs revealed a significant difference between SeNPs-treated infected fruits (**T3**) and untreated infected fruits (**T4**). Likewise, Salem et al. (2022a) reported that 0.5% and 1.0% from SeNPs-composite led to 84.6% and 97.2% reduction of *A. alternata* growth on persimmon fruit, respectively. In addition, Salem et al. (2022b) reported that the treatment with SeNPs-composite for 10 h was effective to decompose the fungus *Penicillium digitatum*.

### Effect of storage temperature on disease progress and shelf-life of tomato fruits

The shelf-life of fruits is determined based on their appearance and spoilage. When 50% of fruits showed symptoms of shrinkage and/or spoilage, the fruits was considered to have reached the end of its shelf-life (Tolasa et al. 2021). In general, ripened fruits are more susceptible to pathogen infection and decay faster than green ones (Rodrigues and Kakde 2019). The main bioactive compounds in ripened tomato are flavonoids, lycopene, and carotenoids as well as soluble sugars, β-carotene, vitamins, and tomatine. During maturation, flavonoids accumulate while the chlorophyll is decreased. However, in green fruits, the content of α-tomatine is higher (500 mg/kg) as compared to ripened red ones (5 mg/kg), which is known to provide protection against pathogens (Chaudhary et al. 2018). Therefore, *Fusarium* infects ripened red tomato fruits causing its rot while using its ascorbic acid and soluble sugars necessary for growth (Bakar et al. 2013).

In general, storage at high temperature fasten the rate of ripening, thus fastening the rate of fruit deterioration, therefore using coolers slows the rate of ripening and extend fruit’s shelf life (Abiso et al. 2015). Tolasa et al. (2021) reported that mature green tomato fruits coated with cactus mucilage can be stored for three weeks. Similarly, Abiso et al. (2015) reported that tomato fruits decay of 16.66% starts early on day 6 for those stored at room temperature and then the decay was raised to 70% on the 12th day. Also, Melkamu et al. (2009) reported that mature green tomato fruits can be stored for 16 days at room temperature. Overall, the treatment of tomato fruits with SeNPs gives an alternative approach for prolonging post-harvest shelf life and maintaining the quality of Cherry tomato up to 25 days at 25 °C.

### Cytotoxicity assessment of SeNPs

The low cytotoxic effect of biosynthesized SeNPs is probably due to several functional biomolecules found in the bio-extract used for biosynthesis and stabilization as confirmed by FTIR analysis, such as O–H, N–H, C–N, C–H, N–O, C–N–C, and C=O. Many researchers suggested that the cytotoxic effect of NPs depends on various factors, such as its administration routes, size, aggregation; time exposure and/or concentration, as well as capping agent used for the stabilization of produced NPs (Tayel et al. 2017; Sorour et al. 2019; Elnady et al. 2022a, b). Similarly, Elnady et al. (2022b) reported lower cytotoxicity on normal cell lines for bio-mediated AgNPs using *Ulva fasciata* and *Citrus japonica* bio-extracts as compared with chemically-synthetized ones. Remarkably, SeNPs have reduced the cytotoxicity toward higher organisms e.g., human and animals within allowed limits, but are highly bioactive against microorganisms, providing many applications in biomedical and nutritional fields (Huerta-Madroñal et al. 2021). Therefore, green bio-mediated SeNPs were used as preservatives for crops, meat products and in anticancer formulations (Salem et al*.* 2022a, b).

### Chemical and physical analysis of tomato fruits

The overall results indicate that post-harvest storage temperature as well as SeNPs treatment affect the characteristics of fresh tomato. However, the treatment of tomato fruits with SeNPs at its MIC positively affected the chemical properties of tomato fruits including TA%, pH, total carotenoids and lycopene, as well as decreased the physiological weight loss %. This effect is probably due to the barrier effect of SeNPs acting against microbial growth and preserving freshness, thus increasing fruits’ shelf life up to 25 and 35 days when stored at 25 °C and 5 °C, respectively.

In the current study, the treatment of tomato fruits with MIC of SeNPs increased the TA% as compared to untreated ones (**T1**), which will probably increase the shelf life of treated tomato fruits. The TA% is generally decreased by increasing the transportation time and repeated vibration, which increases the rate of respiration and consumption of organic acids (Al‐Dairi et al. 2021c). In addition, Endalew (2020) reported that the TA% of tomato was decreased at 25 °C due to the enhancement of tomato ripening and enzymes activity, thus affecting the fruits’ acidity. Also, Al‐Dairi et al. (2021c) reported that the storage of tomato fruits at 22 °C accelerated the TA% reduction after transportation for longer distance. But when fruits were stored at 10 °C, its TA% was increased to 0.31% and 0.29% for short and long distances, respectively, after 12 days.

For total lycopene and β-carotene, it is generally increased with fruits’ ripening and during the storage period (Al‐Dairi et al. 2021a). The development of carotenoid is rapid in tomato stored at room temperature, while it was observed to be slow for those stored in coolers. Tomato fruits stored at 22 °C had 5-folds increase in their total lycopene and carotenoids after 12 days (Al‐Dairi et al. 2021a), but when stored at 10 °C it was increased by 3.5-folds only, this increase was probably due to the accumulation of lycopene and carotenoids resulting from chloroplast conversion to chromoplast (Abiso et al. 2015). In general, harvested tomato at light-red ripening stage have a shorter shelf life as compared to those harvested at earlier stage. In addition, Opara et al. (2012) reported that the lycopene content of ‘Cherry’ cultivar ranged from 6.2 to 56.1 mg/100 g FW during ripening and was increased by 40.7% during ripening. The increase in tomato TA% when stored at 25 °C is probably due to the fungal infection which increased the acidity of infected fruits as previously reported by Jiao et al. (2022) who suggested that organic acids (e.g. citric acid, gluconic acid, or oxalic acid) were secreted by post-harvest fungi, such as *Penicillium* spp. and *Botrytis cinerea,* which are important virulence factors.

For the pH of fresh fruits, generally, it depends mainly on its organic acid contents, and it increases with the increase of storage duration, ripening, and respiration (Endalew 2020). In agreement with our results, the pH value was reported to range from 3.5 to 4.2 for ‘Cherry’, ‘Monika’, and ‘Isabella’ tomato during fruits’ maturation and ripening (Opara et al. 2012). Similarly, Teka (2013) reported that full ripe tomato fruits stored at 22 °C had a higher pH (4.63) as compared to mature green ones stored at 10 °C that had pH of 4.23.

Remarkably, the weight loss % was significantly decreased by increasing the MIC used for treatment, where treated fruits with SeNPs maintained their appearance, color, and weight during their storage at 25 °C and 5 °C for 25 and 35 days, respectively. Likewise, Al-Dairi et al. (2021b) reported that the weight loss in tomato fruits was increased from 3.5 to 6.91% when fruits were stored at 10 °C and 22 °C respectively, for 12 days. It is proposed that SeNPs exhibit antimicrobial activity, thus forming barriers against fungal infection, as well as protecting tomato fruits from moisture loss, managing respiration and over-ripening of treated fruits (Huang et al. 2021; Kumar and Prasad 2021). In addition, Abiso et al. (2015) reported an increase in weight loss (18.36%) of light-red tomato fruits stored at 25 °C for 10 days, as compared to 4.08% when stored in coolers.

Overall, the treatment of tomato fruits with SeNPs at its MIC positively affected the chemical properties of tomato fruits, as well as decreased its weight loss %, confirming the positive barrier effect of SeNPs against *Fusarium* infection as well as preserving freshness, thus increasing the shelf life of fruits. Therefore, SeNPs treatment gives an alternative approach for prolonging shelf-life, maintaining the quality of tomato and providing protection from post-harvest fungal invasion. In addition, biomediated SeNPs is eco-friendly valuable alternative to chemical fungicides in terms of health and food safety.

## Data Availability

Available upon request.
